# Use of angiotensin converting enzyme inhibitors and angiotensin receptor blockers associated with lower risk of COVID-19 in household contacts

**DOI:** 10.1371/journal.pone.0247548

**Published:** 2021-03-02

**Authors:** Katrina Armstrong, Alex Soltoff, Meghan Rieu-Werden, Joshua Metlay, Jennifer Haas

**Affiliations:** Department of Medicine, Massachusetts General Hospital, Boston, Massachusetts, United States of America; University of British Columbia, CANADA

## Abstract

**Background:**

Use of angiotensin converting enzyme inhibitors (ACEI) and angiotensin receptor blockers (ARB) has been hypothesized to affect COVID-19 risk.

**Objective:**

To examine the association between use of ACEI/ARB and household transmission of COVID-19.

**Methods:**

We conducted a modified cohort study of household contacts of patients who tested positive for COVID-19 between March 4 and May 17, 2020 in a large Northeast US health system. Household members were identified by geocoding and full address matching with exclusion of addresses with >10 matched residents or known congregate living functions. Medication use, clinical conditions and sociodemographic characteristics were obtained from electronic medical record (EMR) data on cohort entry. Cohort members were followed for at least one month after exposure to determine who tested positive for SARS-CoV-2. Mixed effects logistic regression and propensity score analyses were used to assess adjusted associations between medication use and testing positive.

**Results:**

1,499 of the 9,101 household contacts were taking an ACEI or an ARB. Probability of COVID-19 diagnosis during the study period was slightly higher among ACEI/ARB users in unadjusted analyses. However, ACEI/ARB users were older and more likely to have clinical comorbidities so that use of ACEI/ARB was associated with a decreased risk of being diagnosed with COVID-19 in mixed effect models (OR 0.60, 95% CI 0.44–0.81) or propensity score analyses (predicted probability 18.6% in ACEI/ARB users vs. 24.5% in non-users, p = 0.03). These associations were similar within age and comorbidity subgroups, including patients with documented hypertension, diabetes or cardiovascular disease, as well as when including other medications in the models.

**Conclusions:**

In this observational study of household transmission, use of ACEIs or ARBs was associated with a decreased risk of being diagnosed with COVID-19. While causality cannot be inferred from these observational data, our results support current recommendations to continue ACEI/ARB in individuals at risk of COVID-19 exposure.

## Introduction

Understanding the risk factors for developing COVID-19 infection after exposure to SARS-CoV-2 is a clinical and public health priority. Multiple studies have found that older age and chronic medical conditions, primarily obesity, hypertension and diabetes, are associated with increased mortality from COVID-19 [1–4]. However, associations with other risk factors, including chronic medication use, have been inconsistent across studies to date.

Use of medications that affect angiotensin converting enzymes (ACE) has received particular attention as a potential risk factor for COVID-19 infection [5–15]. Angiotensin converting enzyme 2 (ACE2) plays a central role in the entry of the SARS-CoV-2 virus into human cells [16, 17]. ACE inhibitors (ACEI) and angiotensin receptor blockers (ARB) have been shown to upregulate ACE2 in animal models [18], and might theoretically increase risk of infection. Multiple studies have have examined the association between ACEI/ARB use and severity of COVID-19 or the risk of COVID-19 test positivity or hospitalization they have been limited by the inability to assess exposure to SARS-COV-2, raising concern about differences in exposure across groups. A single study of nursing home residents exposed to COVID-19 in Belgium suggested that use of ACEI may be protective for infection but the sample size was small and the associations were not statistically significant [19]. To date, there are no published studies examining use of ACEI/ARB and risk of COVID-19 among a large cohort of individuals exposed to SARS-CoV-2. Given this background, we examined the association between use of ACEI/ARB and risk of COVID-19 diagnosis among individuals who lived at the same location as a patient diagnosed with COVID-19 within a large health system population in the Northeastern United States.

## Methods

### Study design

We used a modified cohort study design where cohort members were included at the time of a household member testing positive for SARS-CoV-2 (index case) and followed until they had a positive test for SARS-CoV-2 or until June 16, 2020, one month after the date of the last index case diagnosis. The study was approved by the IRB at MassGeneral Brigham HealthCare, which waived the requirement for informed consent.

### Participants

Index cases were defined as all patients with a positive viral polymerase chain reaction test for SARS-CoV-2 between March 4, 2020 and May 17, 2020 within the MassGeneral Brigham (MGB) Health system (n = 8,672). Home address geocoding was used to match these individuals to other patients registered in the system living at the same address. To maximize the accuracy of identifying household members, geocoding was supplemented with matching on full address information including apartment numbers and addresses identified as correctional facilities, group homes or senior care facilities or with 11 or more matched residents in our data were excluded.

The first household member with a positive test for SARS-CoV-2 was categorized as the index case in the household and excluded from the analyses. If multiple household members tested positive on the same day, the index case was selected randomly. To maximize the accuracy of the electronic medical record (EMR) data for measuring medication use, we excluded household contacts who had not had a documented clinical encounter in MGB in the prior two years, resulting in a final cohort of 9,101 household contacts.

### Study variables

The primary outcome was a positive viral polymerase chain reaction test for SARS-CoV-2 in the MGB Enterprise Data Warehouse (EDW). Given that MGB guidelines largely restricted testing to patients with symptoms during the study period, we categorize a positive test as a diagnosis of COVID-19 recognizing that some infected household members may have been minimally symptomatic and therefore not diagnosed with COVID-19. Because of challenges with test sensitivity early in the study period, seven household contacts were diagnosed with COVID-19 based upon infection control algorithms without a positive test.

Clinical and sociodemographic characteristics of study subjects were determined based upon data from the MGB EDW. Medication use was determined based upon the active medication list at the time of enrollment in the cohort and classified using National Drug Codes into drug classes. While the primary focus was on the association with ACEI/ARB use, other anti-hypertensives, diabetes medications, statins, non-steroidal anti-inflammatory drugs, steroids, other immunomodulatory medications and asthma medications were identified and included in secondary analyses. Clinical conditions were determined based upon information in problem lists and billing codes and included hypertension (HTN), diabetes (DM), asthma, COPD, cardiovascular disease (CVD), cancer, liver disease, and renal disease (S1 Table). Several comorbidities (hemiplegia, dementia, rheumatic disease, peptic ulcer disease, and HIV) were collapsed into a single variable as less than 1% of the study population had any one of these documented comorbidities. Seventy-nine percent of the cohort had both weight and height information documented within two years of study entry, allowing the calculation of body mass index, which was classified into obese and non-obese.

Age was categorized into five groups in years: 19–29.9, 30–49.9, 50–64.9, 65–74.9, and > = 75. Race/ethnicity and limited English proficiency are assessed by self-report at patient registration. Race/ethnicity was categorized as African-American, Asian, Hispanic, White and other. Because recommendations for infection control (e.g., masking, stay at home orders) changed over the study period, time period of diagnosis was categorized into quintiles (March 4 to April 3, April 4 to April 14, April 15 to April 21, April 22 to April 30, May 1 to May 17) and included in the models.

### Statistical analysis

Analyses were conducted using STATASE 16.1. Chi-square and t-tests were used to compare clinical and sociodemographic characteristics between patients taking and not taking ACEI/ARB). The primary analysis used mixed effects logistic regression to assess the relationship between ACEI/ARB use and COVID-19 diagnosis adjusted for potential confounders. Mixed effect models included fixed effects for individual level covariates and a random household effect to adjust for household effects. Propensity scores analyses were used in secondary analyses to assess the robustness of the results. Propensity scores, or the probability of each study subject taking ACE/ARB, were estimated using logit models including all individual level covariates. A nearest neighbor one-to-one matching approach was used to estimate the risk difference between ACE/ARB users and non-users with trimming of the tails of propensity score distribution to minimize the effect of unmeasured confounders. Standardized mean differences were used to assess the success of matching for adjusting for differences in covariates between ACE/ARB users and non-users. Given that several covariates remained imbalanced between groups, mixed effects models were used to examine the association between ACE/ARB use and COVID-19 diagnosis adjusting for propensity score and variables that remained imbalanced after matching. Sensitivity analyses examined subgroups of patients with: (1) diagnosis of HTN, DM or CVD; (2) age of 65 or older; and (3) available BMI data, as well as examining associations between COVID-19 and other medication classes, including beta-blockers, statins, and hypoglycemics.

## Results

Of the 9,101 household contacts, 1,499 were taking an ACEI or an ARB at entry into the study cohort. The characteristics of study subjects by ACEI/ARB use are shown in Table 1. Individuals taking ACEI/ARB were older, less likely to be male, less likely to be Hispanic, and more likely to have comorbid conditions. Level of English proficiency and time period of index case diagnosis did not differ between ACEI/ARB users and non-users. Individuals taking ACEI/ARBs were slightly more likely to be diagnosed with COVID-19 (18.7% vs 15.0%) than individuals not taking those medications in unadjusted analyses, likely reflecting their older age and greater burden of comorbid conditions.

**Table 1:** Participant characteristics.

		ACEI/ARB	No ACEI/ARB	p-value
		n = 1,499	n = 7,602	
		%	%	
Age Category	19–29.9	1.3	31.5	0.0
	30–49.9	11.7	34.6	
	50–64.9	40.9	23.1	
	65–74.9	24.2	6.1	
	> = 75	22.0	4.7	
Gender (male vs female)		49.9	55.7	0.0
Race/ethnicity	White	52.4	40.6	0.0
	Asian	3.7	3.4	
	African American	13.9	12.7	
	Hispanic	23.6	31.8	
	Other	6.3	11.5	
Limited English Proficiency		29.5	30.2	0.6
Clinical conditions	Obesity	48.3	34.2	0.0
	Diabetes	41.8	6.3	0.00
	Hypertension	73.9	4.7	0.00
	Asthma	28.3	13.1	0.00
	COPD	10.9	4.2	0.00
	CVD	16.1	1.6	0.00
	Cancer	9.7	2.2	0.00
	Liver Disease	5.0	1.4	0.00
	Renal Disease	9.2	0.9	0.00
	Other	5.3	1.7	0.00
Time Period	March 4-April 3	22.2	21.7	0.1
	April 4- April 14	22.0	18.6	
	April 15-April 21	17,4	20.9	
	April 22- April 30	19,2	21.0	
	May 1- May 17	19.2	17.9	
Diagnosed with COVID-19		18.7	15.0	0.0

The results of the multivariable analyses are shown in Table 2. After adjustment for age, gender, race/ethnicity, English proficiency, comorbid conditions, and time period, use of ACEI/ARB was associated with a significantly decreased risk of being diagnosed with COVID-19 (OR 0.60, 95% CI 0.44–0.81). Similar associations were found when including ACE and ARB use as separate categories in the model (ACE OR 0.60, 95% CI 0.44–0.82, p = 0.001; ARB 0.58 95%CI 0.40–0.84, p = 0.004). Use of ACE/ARB remained associated with a decreased risk of COVID-19 infection in propensity score analyses with a predicted probability of COVID infection of 18.6% in ACE/ARB users compared to 24.5% in non-users (T-statistic -2.07, p = 0.03). Although covariates were generally well balanced between groups (S2 Table), there were statistically significant mean differences in race, presence of CVD or cancer, and time period of index infection. However, the association between ACE/ARB use and COVID-19 infection remained highly significant after adjusting for the propensity score and these variables (OR 0.65, 95% CI 0.48–0.87, p = 0.00) (S3 Table).

**Table 2:** Associations between ACEI/ARB Use and COVID-19 infection among household contacts of patients with COVID-19.

		Odds Ratio	p-value	95% CI	
ACEI/ARB		0.60	0.00	0.44	0.81
Age	19–29.9	Reference			
	30–49.9	1.71	0.00	1.38	2.13
	50–64.9	2.10	0.00	1.66	2.67
	65–74.9	1.72	0.00	1.24	2.39
	> = 75	1.59	0.01	1.10	2.30
Race/ethnicity	White	Reference			
	Asian	1.72	0.02	1.09	2.72
	African American	1.36	0.03	1.03	1.80
	Hispanic	2.49	0.00	1.95	3.16
	Other	1.86	0.00	1.38	2.52
Male vs female		0.83	0.02	0.72	0.97
Limited English Proficiency		1.36	0.00	1.11	1.66
Comorbid conditions	Hypertension	1.85	0.00	1.40	2.44
	CVD	1.61	0.02	1.09	2.38
	Diabetes	1.59	0.00	1.25	2.03
	Obesity	1.24	0.02	1.03	1.48
	Asthma	1.35	0.01	1.07	1.70
	COPD	1.04	0.82	0.73	1.50
	Cancer	1.63	0.01	1.10	2.41
	Liver Disease	1.60	0.06	0.99	2.58
	Kidney Disease	1.38	0.19	0.85	2.24
	Other	1.82	0.01	1.14	2.88
Time period	March 4-April 3	Reference			
	April 4- April 14	0.85	0.22	0.66	1.10
	April 15-April 21	0.92	0.56	0.70	1.21
	April 22- April 30	0.93	0.60	0.71	1.21
	May 1- May 17	0.67	0.00	0.51	0.88

Sensitivity analyses were conducted to examine the robustness of the results across subgroups and when including use of other medications in the models. (Table 3) The strength of the association between ACE/ARB use and COVID-19 infection was similar across subgroups, although the association was no longer statistically significant in several of the subgroup analyses. The strength of the association increased slightly when adjusting for the use of other medications and remained significant across subgroups except for patients 65 to 74 years of age (p = 0.14). No other medication classes were associated with a reduction in the risk of COVID-19, including beta-blockers, calcium channel blockers, other hypertension medications, nonsteroidal anti-inflammatory agents, statins, asthma medications, steroids and other immunomodulating agents and diabetes medications (S4 Table).

**Table 3:** Sensitivity analyses.

		In models without other medications				In models adjusting for other medications*			
		OR	p-value	95% CI		OR	p-value	95% CI	
Overall		0.60	0.00	0.44	0.81	0.54	0.00	0.39	0.73
In subgroups									
Age	50–64 (n = 2,338)	0.62	0.05	0.39	1.00	0.57	0.03	0.34	0.96
	65–74 (n = 872)	0.64	0.22	0.32	1.30	0.61	0.14	0.31	1.18
	75+ (n = 672)	0.62	0.06	0.37	1.03	0.45	0.01	0.26	0.79
Diagnosis of DM, HTN or CVD (n = 1,896)		0.70	0.06	0.49	1.02	0.65	0.02	0.44	0.94
With BMI information (n = 7,097)		0.64	0.01	0.47	0.89	0.59	0.00	0.42	0.82

*Included B Blockers, Calcium Channel Blockers, Diuretics, Alpha Blockers, Statins, Asthma/COPD Medications, Diabetes Medications, NSAIDs, Steroids, Other Immunomodulatory Medications.

## Discussion

Variation in the risk and severity of COVID-19 infection has been widely described but is poorly understood. Given the observed association between certain chronic diseases and infection risk as well as potential biological mechanisms linking drug actions to disease risk and outcome, the potential for ACEI and ARB to increase COVID-19 risk has been widely debated. As randomized trials of these medications and COVID-19 risk can be challenging to complete within the timeline of the pandemic, making observational studies an important tool for identifying potential associations and informing clinical decision making. To our knowledge, this is the first study to examine the association between use of ACEI and ARB and risk of COVID-19 infection among a large cohort of household contacts of patients with a positive test for SARS-CoV-2.

Use of ACEI/ARB was associated with a higher risk of infection in unadjusted analyses but a lower risk of infection after adjusting for potential confounders and in key subgroups. Most prior studies have been limited to examining severity of disease among patients with COVID-19 and have generally found either no change or a reduction in risk of adverse outcomes among ACEI/ARB users [6, 8, 10, 15, 19]. Studies among patients with hypertension, patients tested for COVID-19 or the general population have also found no association or a protective association between ACEI/ARB use and COVID-19 test positivity, hospitalization or mortality [5–7, 9, 11, 14]. However, these studies have been unable to adjust for exposure to SARS-CoV-2, a key potential confounder, Furthermore, studies of hospitalization or mortality from COVID-19 are unable to differentiate between associations with the risk of transmission and associations with the severity of disease after transmission. Very few previous studies have focused on the risk of COVID-19 transmission in households or other settings. One small study of residents in nursing homes experiencing COVID-19 outbreaks also found that use of ACEI was positively associated with residents being asymptomatic and negatively associated with severe clinical outcomes although neither association reached statistical significance [19]. The current analysis adds to this literature by was able to include a wide range of covariates, which may have further addressed potential confounders that could have masked an association, including use of other medications.

Concern about COVID risk and ACEI/ARB use arose because of evidence that ACEI/ARB increase ACE2 expression in animal models and ACE2 is involved in the entry of SARS-CoV-2 into cells.^16^ However, viral entry also requires transmembrane protease serine 2 (TMPRSS2) which is not affected by ACEI/ARB use [17]. Furthermore, the binding of SARS-CoV-2 spike protein to ACE2 has been shown to downregulate ACE2, leading to an overactivation of angiotensin II which has potent vasoconstrictive and proinflammatory effects [20, 21]. Thus, higher levels of ACE2 may be helpful by increasing the conversion of angiotensin II to angiotensin 1–7 and reducing lung injury from angiotensin II [20]. Furthermore, increased ACE2 in mice has been shown to prevent sepsis induced lung injury [22]. Given that testing was largely restricted to symptomatic patients in the study period, it is possible that the apparent protective effect of ACEI/ARB is driven by a lower risk of becoming symptomatic when infected, rather than a lower risk of infection overall. In either case, these results support the current clinical recommendations that use of ACEI or ARB should not be discontinued because of concern about COVID-19.

These results should be interpreted within the context of study limitations. Individuals were classified as a household contact if they lived at the same address as someone with a positive test. Although we matched on apartment number and excluded addresses with more than ten residents or known group living functions, it is likely that some individuals in the cohort were not in the same household as the person with the positive test during the study period. However, such misclassification is unlikely to be associated with use of ACE/ARBs thereby making it more difficult to have found the observed associations. In addition, household members were not included if they were evaluated in another health system, which is unlikely to be associated with medication use. It is likely that households differed in their infection control practices and while we included a household level effect in the mixed effect models this may not have fully adjusted for unmeasured differences across households. However, it is unlikely that such differences would be correlated with the prevalene of ACE/ARB use within the household. The primary study outcome, testing positive for SARS-CoV-2, required obtaining a test and some patients may not have come in for testing during that time period, particularly if they were less symptomatic. While this limits the generalizability of these results to other outcomes, like risk of asymptomatic or mild infection, it does not affect the relevance of the findings for patients who are sick enough to come in for testing and medical care, arguably the more clinically important outcome. Finally, measurement of medication use through EMR data is well known to be imperfect. However, there is little evidence that such misclassification would be correlated with COVID-19 risk, again making it more difficult to detect associations with medication use. Indeed, analysis of COVID-19 risk associated with other medication use (beta-blockers, statins, hypoglycemics) did not demonstrate a similar protective effect (S4 Table).

In summary, this observational study of household COVID-19 transmission suggests that use of ACE/ARB is associated with a lower risk of becoming symptomatically infected. While these results in no way prove a causal link between medication use and COVID-19 risk, they may be helpful in reassuring patients about continuing ACE/ARB therapy.

## Supporting information

S1 Table Clinical condition coding. (DOCX)

S2 Table Standardized mean differences in matched and unmatched analyses. (DOCX)

S3 Table Mixed effects model of ACE/ARB and COVID-19 infection including propensity score and unbalanced covariates. (DOCX)

S4 Table Associations with other medication classes and COVID-19 diagnosis. (DOCX)
