# Supplementary material for: Use of angiotensin converting enzyme inhibitors and angiotensin receptor blockers associated with lower risk of COVID-19 in household contacts
Source: PLoS One. 2021 Mar 2;16(3):e0247548. doi: 10.1371/journal.pone.0247548 (PMC7924745; doi:10.1371/journal.pone.0247548)
Supplement: S1 Table — (DOCX) [file pone.0247548.s001.docx]

S1 Table. Clinical Condition Coding

|  | ICD-9 | | ICD-10 |
| --- | --- | --- | --- |
| Obesity | 278.00, 278.01, 278.03 | | E66.01, E66.09, E66.1, E66.2,  E66.3, E66.8, E66.9, Z68.1, Z68.20, Z68.21, Z68.2, Z68.23, Z68.24, Z68.25, Z68.26, Z68.27, Z68.28, Z68.29, Z68.30, Z68.31, Z68.32, Z68.33, Z68.34, Z68.35, Z68.36, Z68.37, Z68.38, Z68.39,  Z68.41, Z68.42, Z68.43, Z68.44, Z68.45 |
| Diabetes | 250.0–250.9 | | E10.0-E10.9, E11.0-E11.9, E12.0-E12.9, E13.0-E13.9, E14.0-E14.9 |
| Asthma | 465.9, 466.0, 471.9, 493, 493.00, 493.02, 493.1, 493.10, 493.12, 493.2, 493.22, 493.81, 493.82, 493.9, 493.90, 493.91, 493.92, 648.93, 786.2, E935.3, V12.69, V17.5 | | O99.513, 099.519, J06.9, J20.9, J33.9, J44.1, J44.9, J45.20, J45.21, J45.30, J45.31, J45.40, J45.41, J45.50, J45.51, J45.901, J45.902, J45.909, J45.909, 45.990, J45.991, J45.998, R05, Z82.5, Z87.09, Z88.6 |
| COPD | 416.8, 416.9, 490.x–505.x, 506.4, 508.1, 508.8 | | I27.8, I27.9, J40.x-J47.x, J60.x-J67.x, J68.4, J70.1, J70.3 |
| Hypertension (HTN) | N/A | | 997.91, 401.0, 401.1, 401.9, 402.00, 402.01, 402.10, 402.11, 402.90, 402.91, 403.00, 403.01, 403.10, 403.11, 404.01, 404.02, 404.03, 404.11, 404.12, 404.13, I97.3, I10, I11.9, I11.0, I12.9, I12.0, I13.0, I13.11, I13.2 |
| Cancer | 140.x–172.x, 174.x–195.x, 200.x–208.x, 238.6, 196.x–199.x | | C00.x-C26.x, C30.x-C34.x, C37.x-C41.x, C43.x, C45.x-C58.x, C60.x-C76.x, C77.x-C80.x, C81.x-C85.x, C88.x, C90.x-C97.x |
| Liver Disease | 070.22, 070.23, 070.32, 070.33, 070.44, 070.54, 070.6, 070.9, 570.x, 571.x, 572.2–572.8, 573.3, 573.4, 573.8, 573.9, V42.7, 456.0–456.2 | | B18.x, K70.0-K70.3, K70.4, K70.9, K71.1, K71.3-K71.5, K71.7, K72.1, K72.9, K73.x, K74.x, K76.0, K76.2-K76.4, K76.5, K76.6, K76.7, K76.8, K76.9, Z94.4, I85.0, I85.9, I86.4, I98.2 |
| Kidney Disease | 403.01, 403.11, 403.91, 404.02, 404.03, 404.12, 404.13, 404.92, 404.93, 582.x, 583.0–583.7, 585.x, 586.x, 588.0, V42.0, V45.1, V56.x | | I12.0, I13.1, N03.2-N03.7, N05.2-N05.7, N18.x, N19.x, N25.0, Z49.0-Z49.2, Z94.0, Z99.2 |
| Cardiovascular Disease | 093.0, 362.34, 398.91, 402.01, 402.11, 402.91, 404.01, 404.03, 404.11, 404.13, 404.91, 404.93, 410.x, 412.x, 425.4–425.9, 428.x, 430.x–438.x 437.3, 440.x, 441.x, 443.1–443.9, 447.1, 557.1, 557.9, V43.4 | | G45.x, G46.x, H34.0, I09.9, I11.0, I13.0, I13.2, I21.x-I22.x, I25.2, I25.5, I42.0, I42.5-I42.9, I43.x, I50.x, I60.x-I69.x, I70.x, I71.x, I73.1, I73.8, I73.9, I77.1, I79.0, I79.2, K55.1, K55.8, K55.9, P29.0, Z95.8, Z95.9, |
| Dementia | 290.x, 294.1, 331.2 | | F00.x-F03.x, G30.x, F05.1, G31.1 |
| Rheumatic Disease | 446.5, 710.0–710.4, 714.0– 714.2, 714.8, 725.x | | M05.x, M06.x, M31.5, M32.x-M34.x, M35.1, M35.3, M36.0 |
| Peptic Ulcer | 531.x–534.x | | K25.x - K28.x |
| Hemiplegia or Paraplegia | 334.1, 342.x, 343.x, 344.0– 344.6, 344.9 | | G04.1, G11.4, G80.1, G80.2, G81.x, G82.x, G83.0-G83.4, G83.9 |
| HIV | 042.x–044.x | | B20.x-B22.x, B24.x |
| Medication Categories | | | |
| Diabetes Medications | | repaglinide, nateglinide, miglitol, acarbose, pioglitazone, rosiglitazone, sitagliptin, saxagliptin, alogliptin, linagliptin, glimepiride, glyburide, chlorpropamide, glipizide, tolbutamide, tolazamide, metformin, bromocriptine, colesevelam, dapagliflozin, canagliflozin, empagliflozin, ertugliflozin, pioglitazone, lixisenatide, exenatide, semaglutide, albiglutide, dulaglutide, liraglutide, pramlintide acetate, Insulin glulisine, Insulin aspart, Insulin lispro U-100, Insulin lispro U-200, insulin, NPH insulin, Insulin detemir, Insulin U-100, Insulin glargine U-300, Insulin degludec U-100, Insulin degludec U-200 | |
| Asthma/COPD Medications | | aclidinium bromide, albuterol sulfate, albuterol sulfate, arformoterol tartrate, beclomethasone dipropionate, budesonide, ciclesonide, flunisolide, fluticasone furoate, vilanterol trifenatate, fluticasone propionate, formoterol fumarate, indacaterol, ipratropium bromide, levalbuterol sulfate, mometasone furoate, olodaterol, salmeterol xinafoate, salmeterol xinafoate, tiotropium bromide, umeclidinium | |
| Angiotensin Converting Enzyme Inhibitors | | perindopril, quinapril, ramipril, captopril, benazepril, trandolapril, fosinopril, lisinopril, moexipril, enalapril, enalaprilat, lisinopril | |
| Angiotensin Receptor Blockers | | candesartan, irbesartan, olmesartan, losartan, valsartan, azilsartan, telmisartan, eprosartan, valsartan | |
| Beta Blockers | | nebivolol, timolol, carvedilol, nadolol, propranolol, betaxolol, penbutolol, metoprolol, acebutolol, atenolol, metoprolol, labetalol, pindolol, bisoprolol | |
| Calcium Channel Blockers | | amlodipine, clevidipine, diltiazem, felodipine, isradipine, nicardipine, nifedipine, nimodipine, nisoldipine, verapamil | |
| Diuretics | | spironolactone, torsemide, chlorothiazide, methyclothiazide, hydrochlorothiazide, furosemide, indapamide, hydroflumethiazide, chlorthalidone, metolazone | |
| Alpha Blockers | | doxazosin, phenoxybenzamine, prazosin, terazosin, hydralazine | |
| Statins | | atorvastatin, fluvastatin, lovastatin, pravastatin, rosuvastatin, simvastatin, pitavastatin | |
| Steroids | | budesonide, prednisolone, prednisone | |
| Other Immunomodulatory Medications | | natalizumab, rituximab, cyclosporine, tacrolimus, abatacept, anakinra, ustekinumab, ixekizumab, secukinumab, basiliximab, daclizumab, tocilizumab, leflunomide, mycophenolate, azathioprine, ruxolitinib, tofacitinib, itacitinib, sirolimus, everolimus, adalimumab, certolizumab, etanercept, golimumab, infliximab, vedolizumab | |
| NSAIDS | | celecoxib, diclofenac, diflunisal, etodolac, fenoprofen, flurbiprofen, ibuprofen, indomethacin, ketoprofen, ketorolac, mefenamic acid, meloxicam, nabumetone, naproxen, oxaprozin, piroxicam, sulindac, tolmetin | |
