# Supplementary material for: Use of angiotensin converting enzyme inhibitors and angiotensin receptor blockers associated with lower risk of COVID-19 in household contacts
Source: PLoS One. 2021 Mar 2;16(3):e0247548. doi: 10.1371/journal.pone.0247548 (PMC7924745; doi:10.1371/journal.pone.0247548)
Supplement: S2 Table — (DOCX) [file pone.0247548.s002.docx]

S2 Table. Standardized mean differences in matched and unmatched analyses

|  |  | **Unmatched** | **Matched** |
| --- | --- | --- | --- |
| Age | 19-29.9 |  |  |
|  | 30-49.9 | 0.02 | 0.00 |
|  | 50-64.9 | 0.03 | -0.11 |
|  | 65-74.9 | -0.20 | 0.04 |
|  | >=75 | -0.13 | 0.07 |
| Race/ethnicity | White |  |  |
|  | Asian | -0.57 | 0.00 |
|  | African American | 0.39 | 0.02 |
|  | LatinX | 0.52 | 0.05 |
|  | Other | 0.53 | -0.07 |
|  |  |  |  |
| Gender |  | 0.11 | 0.07 |
| Limited English Proficiency | | -0.02 | 0.02 |
|  |  |  |  |
| Comorbid conditions | HTN | 2.00 | 0.02 |
|  | CVD | 0.53 | 0.09 |
|  | Diabetes | 0.91 | -0.07 |
|  | Obesity | 0.40 | -0.07 |
|  | Asthma | 0.37 | -0.02 |
|  | COPD | 0.25 | -0.04 |
|  | Cancer | 0.32 | 0.16 |
|  | Liver Disease | 0.21 | 0.09 |
|  | Kidney Disease | 0.38 | 0.01 |
|  | Other Comorbidity | 0.19 | -0.10 |
| Time period | March 4-April 3 |  |  |
|  | April 4- April 14 | -0.02 | -0.10 |
|  | April 15-April 21 | 0.00 | -0.09 |
|  | April 22- April 30 | 0.06 | 0.06 |
|  | May 1- May 17 | -0.06 | 0.08 |
