# Supplementary material for: Use of angiotensin converting enzyme inhibitors and angiotensin receptor blockers associated with lower risk of COVID-19 in household contacts
Source: PLoS One. 2021 Mar 2;16(3):e0247548. doi: 10.1371/journal.pone.0247548 (PMC7924745; doi:10.1371/journal.pone.0247548)
Supplement: S3 Table — (DOCX) [file pone.0247548.s003.docx]

S3 Table. Mixed effects model of ACE/ARB and COVID-19 infection including propensity score and unbalanced covariates

|  |  | **OR** | **p-value** | **95% CI** | |
| --- | --- | --- | --- | --- | --- |
| ACE/ARB |  | 0.65 | 0.00 | 0.48 | 0.87 |
| Propensity Score | | 3.89 | 0.00 | 2.62 | 5.78 |
|  |  |  |  |  |  |
| Race/ethnicity | White | Reference | | | |
|  | Asian | 1.71 | 0.02 | 1.11 | 2.65 |
|  | African American | 1.36 | 0.02 | 1.05 | 1.78 |
|  | Hispanic | 2.83 | 0.00 | 2.32 | 3.44 |
|  | Other | 2.19 | 0.00 | 1.67 | 2.88 |
| CVD |  | 1.40 | 0.07 | 0.97 | 2.02 |
| Cancer |  | 1.62 | 0.01 | 1.11 | 2.37 |
| Time period | March 4-April 3 | Reference | | | |
|  | April 4- April 14 | 0.87 | 0.29 | 0.68 | 1.12 |
|  | April 15-April 21 | 0.94 | 0.64 | 0.72 | 1.22 |
|  | April 22- April 30 | 0.95 | 0.72 | 0.74 | 1.23 |
|  | May 1- May 17 | 0.71 | 0.01 | 0.54 | 0.92 |
