# Supplementary material for: Use of angiotensin converting enzyme inhibitors and angiotensin receptor blockers associated with lower risk of COVID-19 in household contacts
Source: PLoS One. 2021 Mar 2;16(3):e0247548. doi: 10.1371/journal.pone.0247548 (PMC7924745; doi:10.1371/journal.pone.0247548)
Supplement: S4 Table — (DOCX) [file pone.0247548.s004.docx]

S4 Table. Associations with Other Medication Classes and COVID-19 Diagnosis

|  | **OR** | **p-value** | **95% CI** | |
| --- | --- | --- | --- | --- |
| Beta blocker | 0.98 | 0.88 | 0.74 | 1.30 |
| Calcium Channel Blocker | 1.40 | 0.03 | 1.03 | 1.90 |
| Other HTN Medication | 0.89 | 0.71 | 0.49 | 1.61 |
| NSAID | 1.56 | 0.00 | 1.29 | 1.88 |
| Statin | 1.28 | 0.06 | 0.99 | 1.67 |
| Asthma Medication | 1.67 | 0.09 | 0.93 | 3.00 |
| Steroid/Immunomodulator | 1.03 | 0.85 | 0.77 | 1.37 |
| Diabetes Medication | 1.47 | 0.19 | 0.83 | 2.63 |
